# Liver disease accompanied by enteropathy in common variable immunodeficiency: Common pathophysiological mechanisms

**DOI:** 10.3389/fimmu.2022.933463

**Published:** 2022-10-20

**Authors:** Fabiana Mascarenhas Souza Lima, Myrthes Toledo-Barros, Venâncio Avancini Ferreira Alves, Maria Irma Seixas Duarte, Cleusa Takakura, Carlos Felipe Bernardes-Silva, Ana Karolina Barreto Berselli Marinho, Octavio Grecco, Jorge Kalil, Cristina Maria Kokron

**Affiliations:** ^1^ Division of Clinical Immunology and Allergy, Hospital das Clinicas HCFMUSP, Faculdade de Medicina, Universidade de Sao Paulo, Sao Paulo, Brazil; ^2^ Department of Pathology, Faculdade de Medicina FMUSP, Universidade de Sao Paulo, Sao Paulo, Brazil; ^3^ Laboratory of the Discipline of Pathology of Transmissible Diseases, Faculdade de Medicina FMUSP, Universidade de Sao Paulo, Sao Paulo, Brazil; ^4^ Department of Gastroenterology, Hospital das Clinicas HCFMUSP, Faculdade de Medicina, Universidade de Sao Paulo, Sao Paulo, Brazil; ^5^ iii-Institute for Investigation in Immunology, Instituto Nacional de Ciência e Tecnologia (INCT), Sao Paulo, Brazil

**Keywords:** inborn errors of immunity (IEI), primary immunodeficiency (PID), common variable immunodeficiency (CVID), portal hypertension, liver disease, nodular regenerative hyperplasia (NRH), enteropathy, duodenal celiac pattern

## Abstract

Common variable immunodeficiency (CVID) is one of the inborn errors of immunity that have the greatest clinical impact. Rates of morbidity and mortality are higher in patients with CVID who develop liver disease than in those who do not. The main liver disorder in CVID is nodular regenerative hyperplasia (NRH), the cause of which remains unclear and for which there is as yet no treatment. The etiology of liver disease in CVID is determined by analyzing the liver injury and the associated conditions. The objective of this study was to compare CVID patients with and without liver–spleen axis abnormalities in terms of clinical characteristics, as well as to analyze liver and duodenal biopsies from those with portal hypertension (PH), to elucidate the pathophysiology of liver injury. Patients were divided into three groups: Those with liver disease/PH, those with isolated splenomegaly, and those without liver–spleen axis abnormalities. Clinical and biochemical data were collected. Among 141 CVID patients, 46 (32.6%) had liver disease/PH; 27 (19.1%) had isolated splenomegaly; and 68 (48.2%) had no liver–spleen axis abnormalities. Among the liver disease/PH group, patients, even those with mild or no biochemical changes, had clinical manifestations of PH, mainly splenomegaly, thrombocytopenia, and esophageal varices. Duodenal celiac pattern was found to correlate with PH (p < 0.001). We identified NRH in the livers of all patients with PH (*n* = 11). Lymphocytic infiltration into the duodenal mucosa also correlated with PH. Electron microscopy of liver biopsy specimens showed varying degrees of lymphocytic infiltration and hepatocyte degeneration, which is a probable mechanism of lymphocyte-mediated cytotoxicity against hepatocytes and enterocytes. In comparison with the CVID patients without PH, those with PH were more likely to have lymphadenopathy (p < 0.001), elevated β_2_-microglobulin (p < 0.001), low B-lymphocyte counts (p < 0.05), and low natural killer-lymphocyte counts (p < 0.05). In CVID patients, liver disease/PH is common and regular imaging follow-up is necessary. These patients have a distinct immunological phenotype that may predispose to liver and duodenal injury from lymphocyte-mediated cytotoxicity. Further studies could elucidate the cause of this immune-mediated mechanism and its treatment options.

## Introduction

Common variable immunodeficiency (CVID) is an inborn error of immunity that has a major clinical impact, especially in adults (1–3). It was recently recognized that CVID comprises a group of diseases now known as CVID disorders. Although most CVID cases are sporadic, monogenic defects are found in 10%–54% (4–6). Patients with CVID present distinct phenotypes, which are associated with different prognoses and mortality rates (7–10). The main clinical manifestations are infections of the upper and lower respiratory tract, which occur in 90% of cases (11). Autoimmune disorders are observed in 25%–30% of patients with CVID, cytopenias being the most common (12–14). In addition, CVID patients can have nonmalignant lymphadenopathy, such as splenomegaly, diffuse lymphadenopathy, nodular lymphoid hyperplasia, and granulomatous disease (7, 13), as well as being at an increased risk for malignancies, especially non-Hodgkin B-cell lymphoma and gastric cancer (13, 15). The reported prevalence of liver disease in CVID ranges from 11.9% to 79.0%, depending on the diagnostic criteria applied. The causes of liver disease in CVID include nodular regenerative hyperplasia (NRH), hepatitis C, autoimmune hepatitis, primary sclerosing cholangitis, and liver cancer. The management of liver disease in patients with CVID poses a challenge. Among patients with CVID, the rates of morbidity and mortality associated with the clinical complications of portal hypertension (PH) secondary to liver disease, such as ascites and esophageal varices, are higher in those with liver–spleen axis abnormalities than in those without (16–18).

The main liver disorder observed in CVID patients is NRH (17, 19–21), which is accompanied by microvasculature perfusion disorders, liver injury, and the consequent regeneration of hepatocytes, with the formation of nodules and compression of adjacent structures, leading to PH (22–25). The cause of NRH is not well understood, and there is no specific treatment for it. There have been reports of NRH in individuals with autoimmune disorders, neoplasia, or HIV infection (26–28). The co-occurrence of NRH and PH is common in CVID (19, 20). Granulomatous liver disease, primary biliary cirrhosis, and hepatitis of unknown origin have also been observed in CVID patients (3, 17, 29). Because the clinical pictures of these pathologies are similar to that of NRH, the cause of noncirrhotic PH can be confirmed only through histopathological analysis.

Crescenzi et al. (30) found that enteropathy and lymphadenopathy showed significant associations with liver disease in CVID, such associations having also been observed for hepatobiliary diseases including noncirrhotic PH, primary biliary cirrhosis, and autoimmune hepatitis (31–33). Diarrhea is common in CVID, occurring in 20%–60% of cases. The main pathogen that causes infectious diarrhea in patients with CVID is *Giardia lamblia*, followed by *Salmonella* spp. and *Campylobacter jejuni* (3, 13, 34–37). Norovirus infection has also been reported as a cause of enteropathy in CVID (38). In CVID, diarrhea of noninfectious causes is due to Crohn’s disease, ulcerative colitis, or duodenal/ileal nodular lymphoid hyperplasia (7, 13, 39). In patients with CVID, duodenal changes mimicking celiac disease (celiac pattern) can occur, although the symptoms rarely improve with gluten exclusion and they can be accompanied by infection (39–41).

Liver disease and PH are common findings in CVID, leading to increased morbidity and mortality, although the causes remain unclear (42). Immunoglobulin replacement therapy does not change the course of liver disease, and there is therefore still no specific treatment for this pathology. A detailed analysis of liver damage, together with characterization of the clinical and immunological conditions associated with PH and liver disease in CVID, is essential to elucidate the etiology and allow better management of cases (43).

## Methods

### Study design and population

In this study, we evaluated a cohort of CVID patients, retrospectively and prospectively, between 1987 and 2021 at the Outpatient Immunology and Allergy Clinic of the *Hospital das Clínicas da Faculdade de Medicina da Universidade de São Paulo* (HC-FMUSP, University of São Paulo School of Medicine *Hospital das Clínicas*), in the city of São Paulo, Brazil. The diagnostic criteria applied were those described by Bonilla et al. (1): presenting with at least one of the characteristic clinical manifestations of CVID (infection, autoimmune disorder, or lymphoproliferative disorder); having hypogammaglobulinemia, as defined according to the age-adjusted reference range for the laboratory at which the measurement is performed; having a low IgG level in at least two measurements made more than 3 weeks apart; having a low level of IgA or IgM; and other causes of hypogammaglobulinemia having been excluded. We included all patients thus diagnosed with CVID during the period of interest, with no restrictions regarding age, sex, or comorbidities. Although patients with PH or liver disease of secondary causes—viral hepatitis (diagnosed by the identification of antigenemia for hepatitis B and on the basis of the serum polymerase chain reaction for hepatitis C), hemochromatosis, Wilson’s disease, alcoholic steatohepatitis, schistosomiasis, occlusive venous disease, or neoplasia—would have been excluded, no such patients were identified.

**Figure 1:**
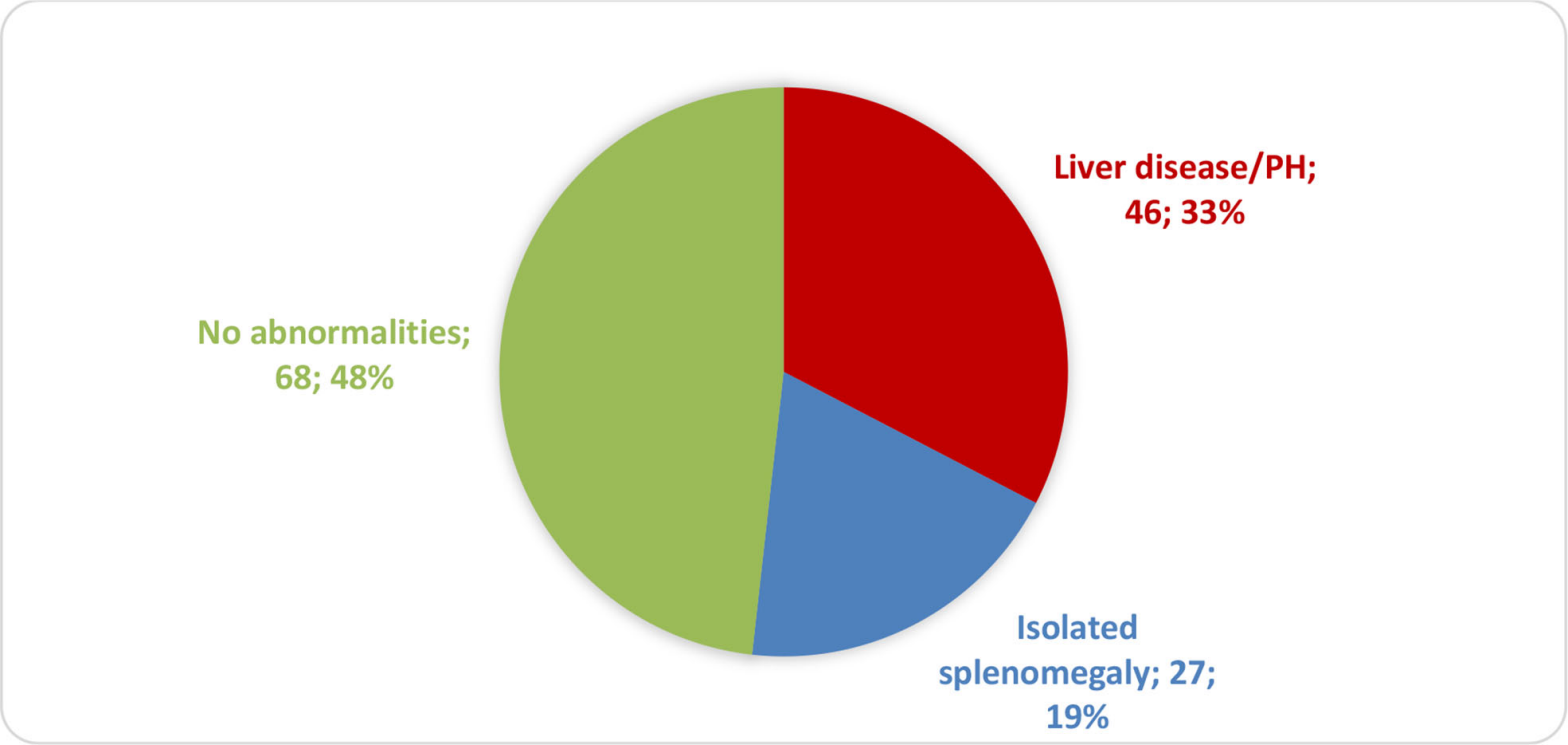
Distribution of CVID patients (***n*** = 141) by liver–spleen axis abnormalities.

Of a total of 208 eligible patients, 67 were excluded because they were lost to follow-up or because there were missing data. The remaining 141 patients were divided into three groups on the basis of their diagnosis (**Figure 1**): liver disease with or without PH (liver disease/PH), isolated splenomegaly, and no liver–spleen axis abnormalities. The project was approved by the HC-FMUSP Research Ethics Committee (Reference no. 94154618.6.0000.0068; Ruling no. 2,827,628). Because the diagnostic tests were performed in the routine follow-up of patients and the clinical data were collected retrospectively, the requirement for informed consent was waived.

### Diagnosis of liver disease, portal hypertension and splenomegaly

For the division of patients into the three subgroups—liver disease/PH, isolated splenomegaly, and no liver–spleen axis abnormalities—pre-established criteria were applied. Noncirrhotic PH was diagnosed as recommended by Verheij et al. (44) and Schouten et al. (45). For PH, chronic liver disease, and splenomegaly (**Chart 1**), the diagnoses were based on the radiological criteria established by the Department of Radiology, HC-FMUSP (46).

**Chart 1:** Diagnostic criteria adopted for PH, liver disease, and splenomegaly.

Diagnosis	Criteria
PH	• Clinical evidence: esophageal varices, hypertensive gastropathy, non-neoplastic ascites, or collateral circulation of the liver–spleen axis **or** • Portal vein diameter > 1.2 cm and splenomegaly or evidence of hypersplenism (e.g., thrombocytopenia)
Liver disease	• Imaging signs of chronic parenchymal liver disease, except fatty infiltration; especially when associated with splenomegaly, hypersplenism, or alterations in hepatocellular or cholestatic liver enzymes for more than 6 months
Splenomegaly	• Spleen >12 cm in length
No liver–spleen axis abnormalities	• Absence of all of the alterations described above

### Liver and duodenal biopsies

Liver and duodenal tissues were collected surgically and by upper gastrointestinal endoscopy, respectively. The procedures were performed in accordance with the routine practice at the HC-FMUSP Outpatient Clinic for Clinical Gastroenterology and Primary Immunodeficiencies.

The indication for liver biopsy in patients with PH was made by the HC-FMUSP Department of Clinical Gastroenterology and was based on pre-established guidelines (44, 45). Biopsy specimens were fixed in formalin and embedded in paraffin, after which 4-µm-thick slices were cut and stained with hematoxylin and eosin, Masson’s trichrome, picrosirius red, or reticulin. All of the stained slides were evaluated by optical microscopy.

To perform duodenal biopsies, upper gastrointestinal endoscopy was performed in accordance with the institutional protocol of the HC-FMUSP Department of Clinical Gastroenterology. Duodenal tissue specimens were fixed in formalin and embedded in paraffin, after which 4-µm-thick slices were cut and stained with hematoxylin and eosin. All of the stained slides were evaluated under light microscopy to identify and characterize any lesions.

### Pathological diagnosis

All biopsies were reviewed by two experienced pathologists specializing in liver diseases. The pathologists made the diagnoses and performed semiquantitative analyses of the histological variables.

In picrosirius red- or reticulin-stained slides, NRH of the liver was defined on the basis of the following criteria (24, 25): regenerative plaques in an area ≥2 hepatocytes with intervening areas of thinned trabeculae and atrophic hepatocytes, absence of bridging fibrosis, or established cirrhosis.

Epithelioid granuloma was defined by the presence of clusters of ≥10 epithelioid cells. Screening for tuberculosis bacilli was performed by Ziehl–Neelsen staining.

The diagnosis of portal vein endotheliitis was based on a finding of permeation of the endothelium by lymphocytes, together with endothelial cell damage (47).

Autoimmune hepatitis was defined according to the following criteria (27): marked interface hepatitis; centrizonal accentuation of inflammation; apoptotic hepatocytes; hepatocyte rosettes; or chronic active hepatitis.

Liver fibrosis was scored as follows (48): 0, no fibrosis; 1, portal fibrosis without septa; 2, portal fibrosis with few septa; 3, incomplete nodular formations; or 4, cirrhosis with nodules. Similarly, inflammation of the liver was scored as follows (48): 0, none; 1, mild; 2, moderate; or 3, intense. Lymphadenopathy was defined as an enlargement of the thoracic or abdominal lymph nodes to ≥1 cm in diameter.

Duodenal changes mimicking celiac disease, called celiac patterns, were defined as villous atrophy, crypt hyperplasia, and intraepithelial lymphocytosis in ≥30% of epithelial cells (41).

For comparison, an inflammatory infiltrate–necrosis–fibrosis score (0, absent; 1, mild; 2, moderate; or 3, severe) was applied in 10 random fields per (liver or duodenal) specimen. The specimens were analyzed under light microscopy at a magnification of ×200.

### Electron microscopy

We analyzed a total of 11 liver biopsies and eight duodenal biopsies. Four liver biopsy specimens and four duodenal biopsy specimens were analyzed by electron microscopy, in accordance with the routine protocol employed in the Laboratory of Pathology of Transmissible Diseases within the FMUSP Department of Pathology. Thus, a fragment of the area of interest was removed from the paraffin block, immersed in xylene for 12 h for deparaffinization, and dehydrated in a graded ethanol series—100% (absolute ethanol) for 15 min, 90% for 15 min, and 70% for 15 min. Each fragment was then washed twice in buffer solution and fixed in 3% glutaraldehyde, subsequently being submitted to rapid processing for electron microscopy. The biopsy material present in the paraffin blocks of the other samples was not sufficient for electron microscopy processing. Liver and duodenal tissues were analyzed. Cellular and structural alterations were identified by comparison with the areas that were categorized as preserved. A lymphocyte count in the area of interest ≥10% higher than that in the preserved areas was classified as a clinically relevant difference.

### Biochemical parameters

Liver-related biochemical parameters were measured in the liver disease/PH group. The frequency of serum platelet measurements below the reference value (thrombocytopenia) was compared among the three groups. Serum levels of β_2_-microglobulin were also compared among the groups.

All liver disease/PH group patients tested negative for autoimmune hepatitis (on anti–smooth-muscle and anti–liver–kidney microsomal antibody tests) and for primary biliary cirrhosis (on an anti-mitochondrial antibody test). The differential diagnosis of these liver pathologies was excluded in the analysis of the liver biopsies, given that autoantibody tests can be inaccurate in patients with hypogammaglobulinemia.

### Peripheral blood immunophenotyping

In all three groups, lymphocytes were quantified in peripheral blood. The lymphocytes of interest were B cells (CD19^+^ cells), T cells (CD3^+^CD4^+^ and CD3^+^CD8^+^ cells), and natural killer (NK) cells (CD3^−^ CD16^+^ CD56^+^ cells).

### Statistical analysis

Clinical, biochemical, and imaging data were collected from patient charts and compared among the three groups. Statistical analyses were performed with Microsoft Excel, the IBM SPSS Statistics software package version 24.0 (IBM Corporation, Armonk, NY, USA), and the GraphPad Prism software version 7.0b for Macintosh (GraphPad Software, Inc., San Diego, CA). For the comparison of categorical variables, Fisher’s exact test was used. For continuous variables, the Kolmogorov–Smirnov and Shapiro–Wilk normality tests were applied. For those with normal distribution, Student’s *t*-test and analysis of variance were used, whereas the Mann–Whitney *U* and Kruskal–Wallis tests were used for those with non-normal distribution. For all tests, a significance level of 0.05 (α = 5%) was adopted. Therefore, values of p < 0.05 were considered significant.

## Results

### Study population

Of the 141 CVID patients included in this study, 46 (32.6%) were in the liver disease/PH group, 27 (19.1%) were in the isolated splenomegaly group, and 68 (48.2%) were in the liver–spleen axis group (**Figure 1**). As shown in **Table 1**, the mean age was higher in the liver disease/PH group than in the other two groups. There were no significant differences among the three groups in terms of sex, disease duration, age at CVID diagnosis, or delay in diagnosis.

**Table 1:** Demographic and clinical characteristics of the CVID patients evaluated (*N* = 141), by group.

Characteristic	NLSA	IE	LD/PH	p
	(*n* = 68)	(*n* = 27)	(*n* = 46)	
Age (years), mean ± SD	44.5 ± 14.9	40.9 ± 13.3	52.8 ± 15.9	0.146 (NLSA vs. IE)
				0.015 (NLSA vs. LD/PH)
				0.001 (IE vs. LD/PH)
Age at diagnosis (years), mean ± SD	30.5 ± 14.1	22.3 ± 10.9	34.5 ± 15.7	0.125 (NLSA vs. IE)
				0.17 (NLSA vs. LD/PH)
				0.053 (IE vs. LD/PH)
Age at symptom onset (years), mean ± SD	17.8 ± 13.7	14.5 ± 11.7*	20.8 ± 1.5	0.242 (NLSA vs. IE)
				0.287 (NLSA vs. LD/PH)
				0.055 (IE vs. LD/PH)
CVID duration (years), mean ± SD	27.6 ± 14.6	26.7 ± 15.5*	31.8 ± 16.4	0.782 (NLSA vs. IE)
				0.164 (NLSA vs. LD/PH)
				0.189 (IE vs. LD/PH)

SD, standard deviation; NLSA, no liver spleen abnormalities; IE, isolated splenomegaly; LD, liver disease.

*n = 26 (data unavailable for one patient).

### Liver disease, portal hypertension and their complications

Only four of the patients in the liver disease/PH group showed signs of chronic, noncirrhotic, non-fatty liver disease without PH: one had splenomegaly and persistent alterations in liver canalicular enzymes; two had splenomegaly, thrombocytopenia, and persistent alterations in liver canalicular enzymes, one of those two also showing an elevated level of direct bilirubin; and one had previously undergone splenectomy due to an external cause and showed no biochemical alterations. Of the 46 patients in the liver disease/PH group, 27 (58.7%) had esophageal or gastric varices: Two with gastric varices only and 25 with both. Splenomegaly was observed in 38 (82.6%) of the liver disease/PH group patients.

Of the 46 patients in the liver disease/PH group, five (10.9%) showed no liver-related biochemical alterations except for thrombocytopenia. It is noteworthy that normal laboratory test results and normal platelet counts were seen in six patients (13.0%). When present, biochemical changes were mild in most cases. We found that aspartate aminotransferase (AST) was elevated (1.2–2.8× the normal value) in 16 patients (34.8%), alanine aminotransferase (ALT) was elevated (1.1–3.1× the normal value) in 14 (30.4%), alkaline phosphatase (ALP) was elevated (1.1–5.1× the normal value) in 28 (60.9%), and gamma-glutamyltransferase (GGT) was elevated (1.1–6.8× the normal value) in 26 (56.5%). Total bilirubin was elevated (1.1–6.3× the normal value) in 10 patients (21.7%), and platelet counts were below the reference value in 29 patients (63.0%). Of the 141 CVID patients in the sample, 39 (27.7%) had thrombocytopenia, which was identified in 29 (63.0%) of the patients in the liver disease/PH group, compared with only five (8.6%) of the 58 patients in the no liver–spleen axis abnormalities group and only five (18.5%) of the 27 patients in the isolated splenomegaly group (**p** < 0.001 for both). Among the 46 patients in the liver disease/PH group, signs of parenchymal liver disease and hepatomegaly were observed on abdominal ultrasound or computed tomography scan in 15 (32.6%) and six (13.0%), respectively. Only two patients showed a significant change in coagulation (international normalized ratio). **Tables S1** and **Table 2** show the clinical and biochemical characteristics of the patients with PH.

**Table 2:** Clinical and biochemical features of the CVID patients diagnosed with liver disease/PH (n = 46)*.

Feature	*n* (%)
Clinical	
Splenomegaly	38 (82.6)
Esophageal varices	18 (39.1)
Gastric varices	9 (19.6)
Hepatomegaly	6 (13.0)
Collateral circulation or abdominal shunt	6 (13.0)
Ascites	4 (8.7)
Biochemical	
AST increased	16 (34.8)
ALT increased	14 (30.4)
ALP increased	28 (60.9)
GGT increased	26 (56.5)
TB or DB increased	10 (21.7)
INR increased	2 (4.3)
Thrombocytopenia	29 (63.0)
Normal labs	6 (13.0)
Normal labs except platelets	5 (10.9)

TB, total bilirubin; DB, direct bilirubin; INR, international normalized ratio.

*Only four patients showed signs of chronic liver disease without PH.

### Duodenal celiac pattern

**Figure 2:**
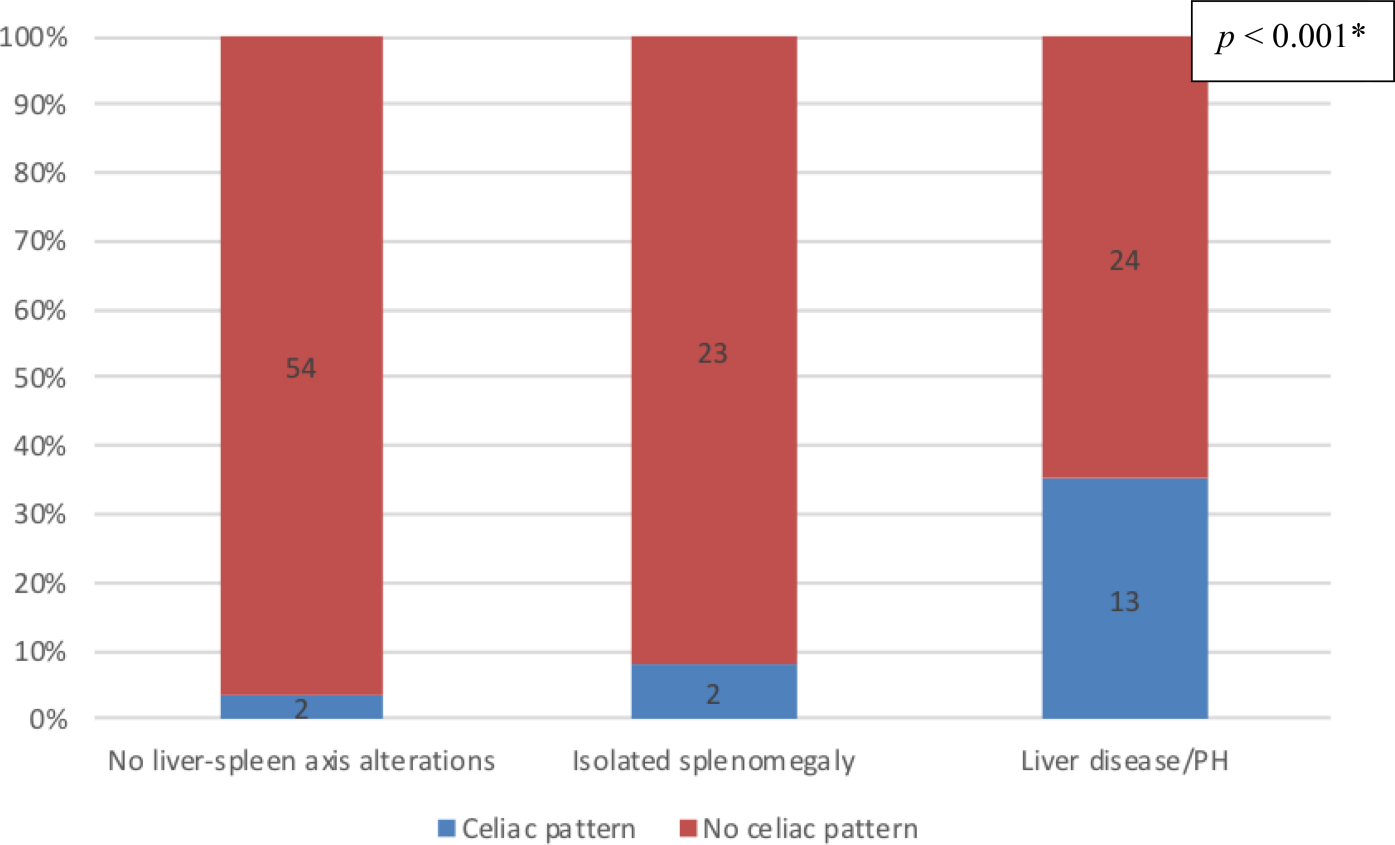
Prevalence of duodenal changes consistent with celiac pattern, by group, among the CVID patients evaluated (*n* = 118); no liver-spleen axis alterations n = 56; isolated splenomegaly n = 25; liver disease/PH n = 37. ^†^ *Versus the other two groups (Fisher’s exact test). ^†^Data unavailable for 23 patients.

Endoscopy findings were available for a total of 118 patients: 37 (31.4%) in the liver disease/PH group; 56 (47.4%) in the no liver–spleen axis abnormalities group; and 25 (21.2%) in the isolated splenomegaly group. As illustrated in **Figure 2**, duodenal celiac pattern (atrophy and intraepithelial lymphocytosis) were present in 17 (14.4%) of the 118 patients analyzed—13 (35.1%) of the 37 in the liver disease/PH group, compared with only two (3.6%) of the 56 in the no liver–spleen axis abnormalities group and two (8.0%) of the 25 in the isolated splenomegaly group (**p** < 0.001 for both). All 17 of those patients had intermittent or chronic diarrhea. The duodenal enteropathy was ameliorated by the adoption of a gluten-free diet in only two patients (both in the liver disease/PH group). In our sample, there was no significant association between giardiasis and duodenal celiac pattern (**p** > 0.05).

### Nodular regenerative hyperplasia, lymphocytosis and duodenal atrophy

**Figure 3:**
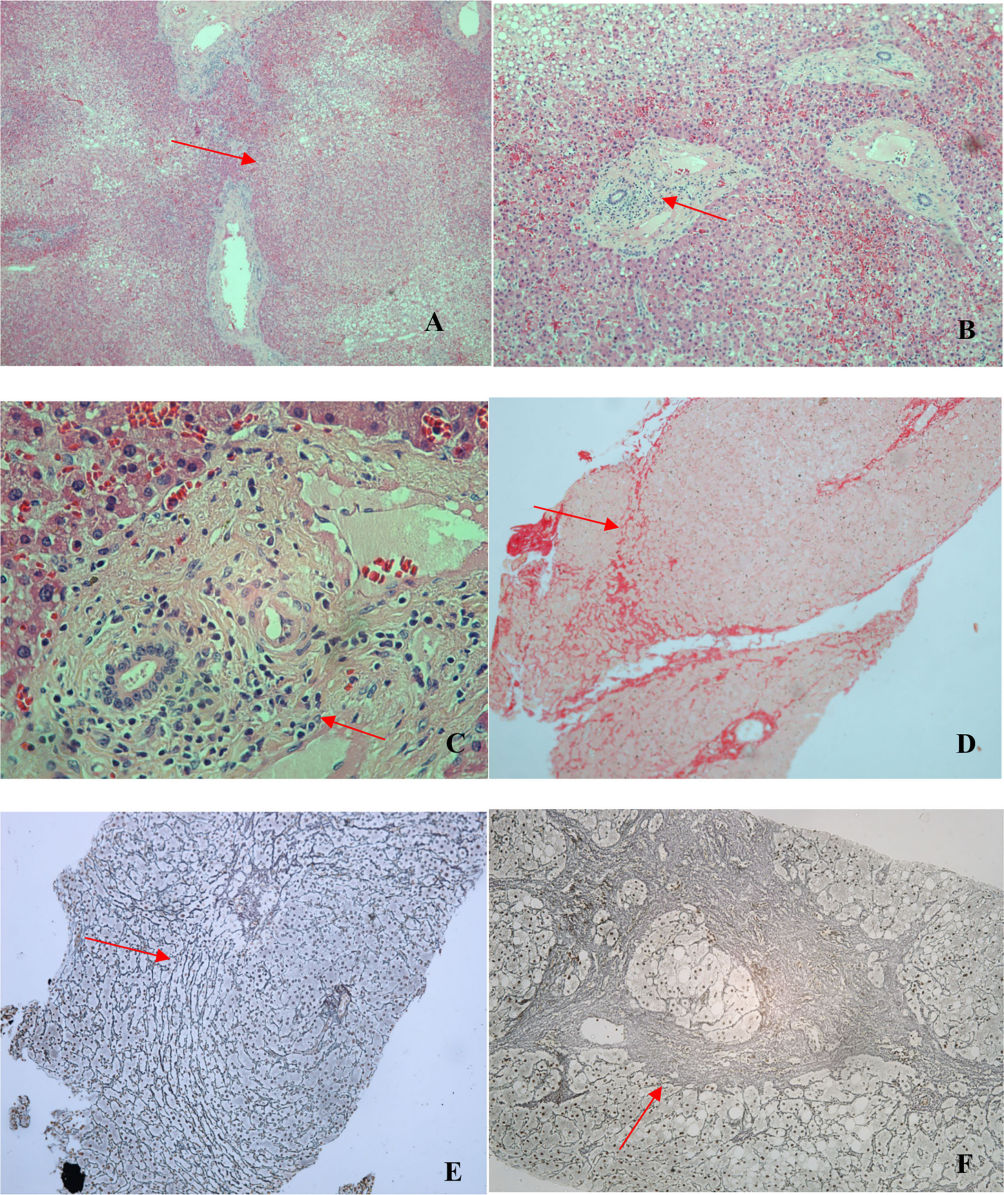
Photomicrographs of liver biopsy specimens from CVID patients. All liver biopsies (*n* = 11) met the criteria for a diagnosis of NRH. **(A)** Nodular formation (arrow; hematoxylin and eosin [H&E]; magnification, ×40). **(B)** Periportal inflammatory infiltrate (arrow; H&E; magnification, ×100). **(C)** Periportal inflammatory infiltrate (arrow; H&E; magnification, ×400). **(D)** Nodular formation and incomplete fibrosis (arrow; picrosirius red; magnification, ×100). **(E)** Nodular formation (arrow; reticulin; magnification, ×100). **(F)** Nodular formation with a focus of marked fibrosis, present in only one patient (arrow; reticulin; magnification, ×100).

Histopathological analysis of the 11 liver biopsies showed nodular formations with hepatocyte atrophy and regeneration, together with mild-to-moderate periportal inflammatory infiltrate, composed mainly of mononuclear cells, in all biopsy specimens (**Figure 3**). Only one specimen showed severe periportal inflammation. Portal macrophages, Kupffer hyperplasia/hypertrophy, and sinusoidal congestion were also observed in four cases. None of the cases showed any relevant distortion of the liver architecture or the lobular plaque, although all of them showed mild or moderate fibrosis, noncirrhotic portal fibrosis, and solitary nodules. All 11 liver biopsies met the criteria for a diagnosis of NRH. Non-alcoholic fatty liver disease was observed in three biopsy specimens, all of which were from patients with NRH.

**Figure 4:**
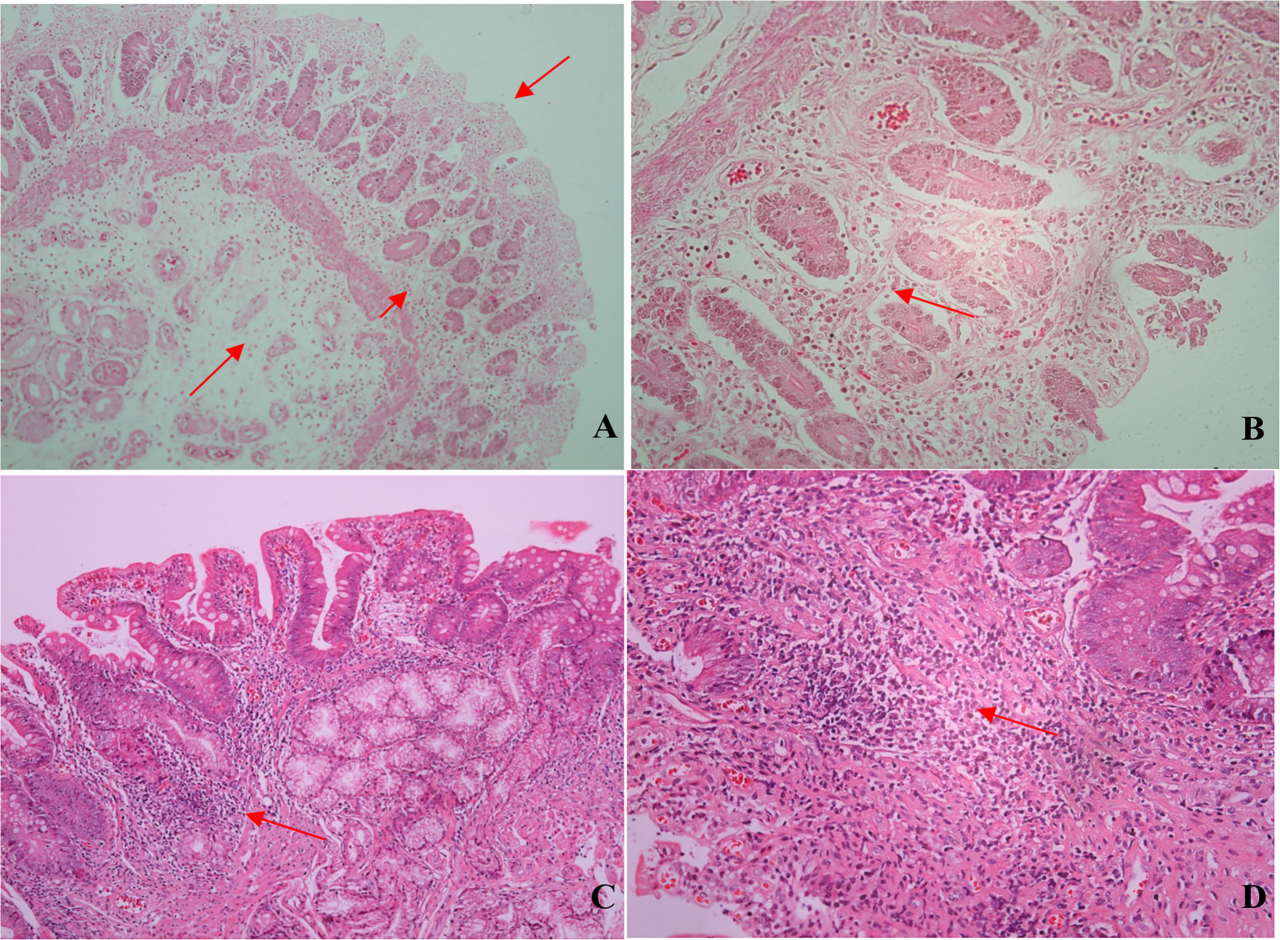
Photomicrographs of duodenal biopsy specimens from CVID patients. **(A)** Mucosal atrophy, edema, and inflammatory infiltrate (arrows; hematoxylin and eosin [H&E]; magnification, ×100). **(B)** Mucosal atrophy, gland regeneration, and mononuclear inflammatory infiltrate (arrow; H&E; magnification, ×200). **(C)** Mononuclear Inflammatory infiltrate (arrow; H&E; magnification, ×100). **(D)** Inflammatory infiltrate: lymphocytes, macrophages, and plasma cells (arrow; H&E; magnification, ×200).

Among the eight duodenal biopsies analyzed (all from patients from the liver disease/PH group), inflammatory infiltrate (composed mainly of mononuclear cells), of varying intensity, was observed in seven and duodenal atrophy was observed in three. We also observed mucosal edema, regeneration of intestinal glands, and, in some cases, macrophages and plasma cells, on a smaller scale (**Figure 4**).

The details of the histopathological analysis of the liver and duodenum are described in **Table 3**. **Table 4** summarizes the histopathological findings.

**Table 3:** Histopathological description of liver and duodenal biopsies from 11 CVID patients with liver disease/PH.

Patient	Diagnosis	Liver inflammation*	Hepatic lymphocytosis*	Liver necrosis*	Noncirrhotic hepatic fibrosis*	Duodenal atrophy*	Duodenal lymphocytosis*	Findings
1	NRH	0	0	0	1	yes	3	Hepatocyte ballooning
								Sinusoidal dilatation
								Macrovesicular and microvesicular steatosis
5	NRH	3	3	0	1	yes	3	Lymphoid aggregates
								Macrophages and plasma cells in the portal space
								Hepatocyte ballooning and degeneration
11	NRH	1	1	1	1	no	1	Lytic necrosis
								Lobular inflammation
15	NRH	1	0	1	1	no	2	Macrophages in the portal space
								Kupffer hyperplasia; ballooning and degeneration of hepatocytes
17	NRH	2	2	0	1	yes	3	Lymphocytosis
								Macrophages and a few plasma cells in the portal space
								Macrovesicular and microvesicular steatosis
21	NRH	2	2	1	2	NA	NA	Lymphocytosis
								Macrophages in the portal space
								Macrovesicular and microvesicular steatosis
24	NRH	2	2	0	3	no	2	Lymphocytosis
								Some macrophages in the portal space
								Kupffer and sinusoidal hypertrophy
35	NRH	1	1	1	1	NA	NA	Portal space lymphocytosis
								Sinusoidal congestion
37	NRH	1	1	0	2	NA	NA	Portal space lymphocytosis
								Sinusoidal congestion
38	NRH	1	1	0	1	no	0	Portal space lymphocytosis
41	NRH	1	1	0	2	no	2	Lymphocytes and macrophages in the portal space
								Congestion

*0 = no inflammation or fibrosis; 1 = mild; 2 = moderate; 3 = severe. NA, not available.

**Table 4:** Summary of the histopathological description of liver biopsies (*n* = 11) and duodenal biopsies (*n* = 8) in patients with liver disease/PH.

Description of the injury	Frequency
Hepatic architecture distortion with nodular formation (*n* = 11)	Present in all cases
Predominantly periportal hepatic lymphocytosis (*n* = 11)	Mild-to-moderate in eight cases, severe in one
Noncirrhotic hepatic fibrosis (*n* = 11)	Mild-to-moderate in 10 cases, severe in one
Hepatocyte necrosis (*n* = 11)	Mild, present in four cases
Macrophages in the portal space and Kupffer hyperplasia (*n* = 11)	In smaller numbers, present in six cases
Sinusoidal congestion (*n* = 11)	Present in four cases
Duodenal lymphocytosis (*n* = 8)	Mild-to-moderate in five cases, severe in two
Duodenal atrophy (*n* = 8)	Present in three cases

### Lymphocyte-mediated cytotoxicity against hepatocytes and enterocytes

**Figure 5:**
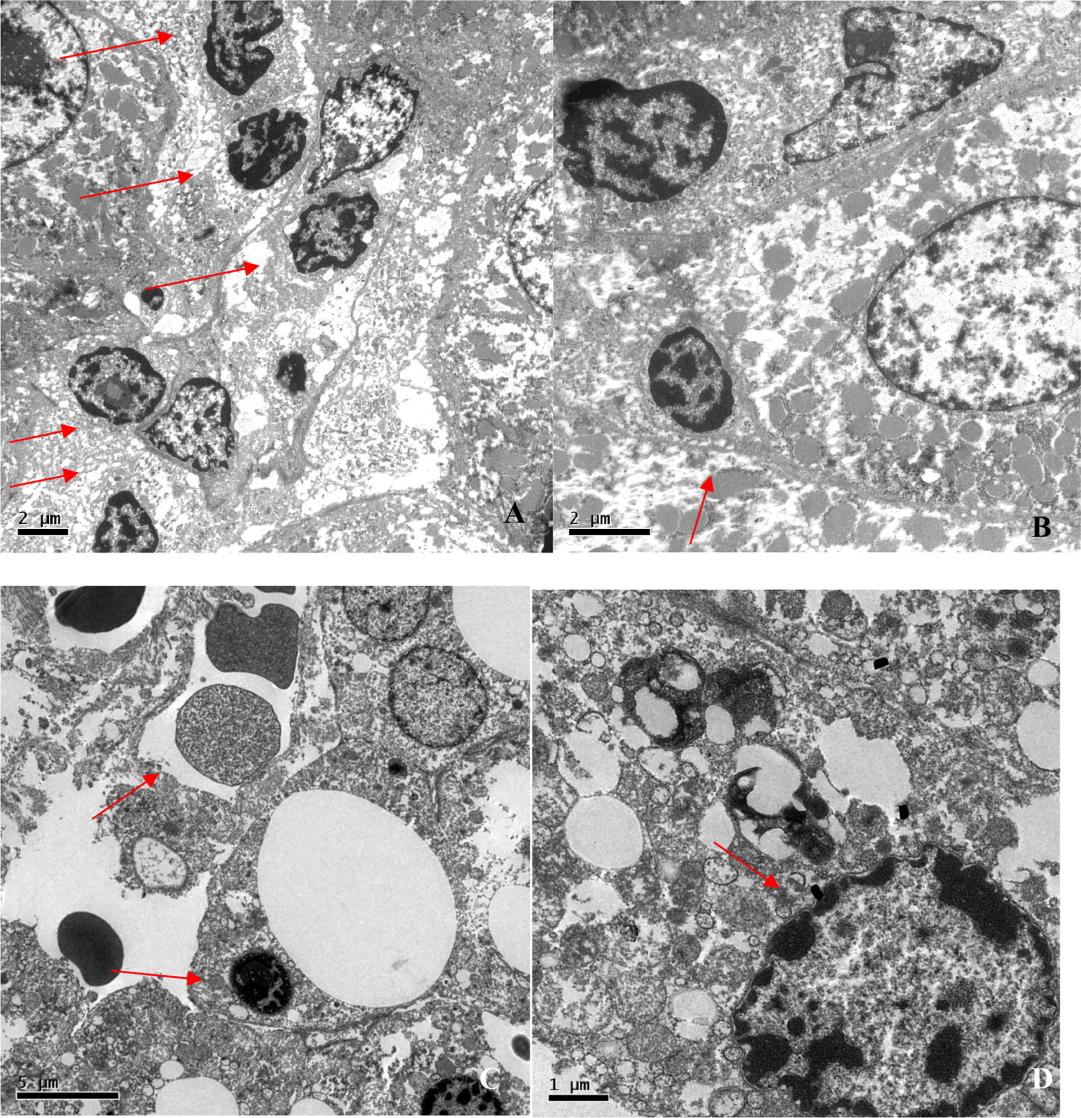
Electron photomicrographs of liver biopsies. **(A)** Lymphocytic infiltration (arrows; magnification, ×6,000). **(B)** Lymphocyte bordering a hepatocyte (arrow; magnification, ×10,000). **(C)** Councilman corpuscle and lymphocytic infiltration (arrows; magnification, ×5,000). **(D)** Nuclear lipofuscin pigment suggesting hepatocyte degeneration (arrow; magnification, ×15,000).

Electron microscopy, which was employed in four liver biopsies, showed mild-to-moderate localized lymphocytic infiltration and hepatocyte degeneration. Lymphocytes were identified by their electron-dense nuclei and cytoplasm, as well as by the fact that, in lymphocytes (especially T lymphocytes), the nucleus is considerably larger than is the cytoplasm, which is not the case in hepatocytes. The images suggest that lymphocyte-mediated cytotoxicity against hepatocytes is the mechanism of liver injury in CVID (**Figure 5**).

**Figure 6:**
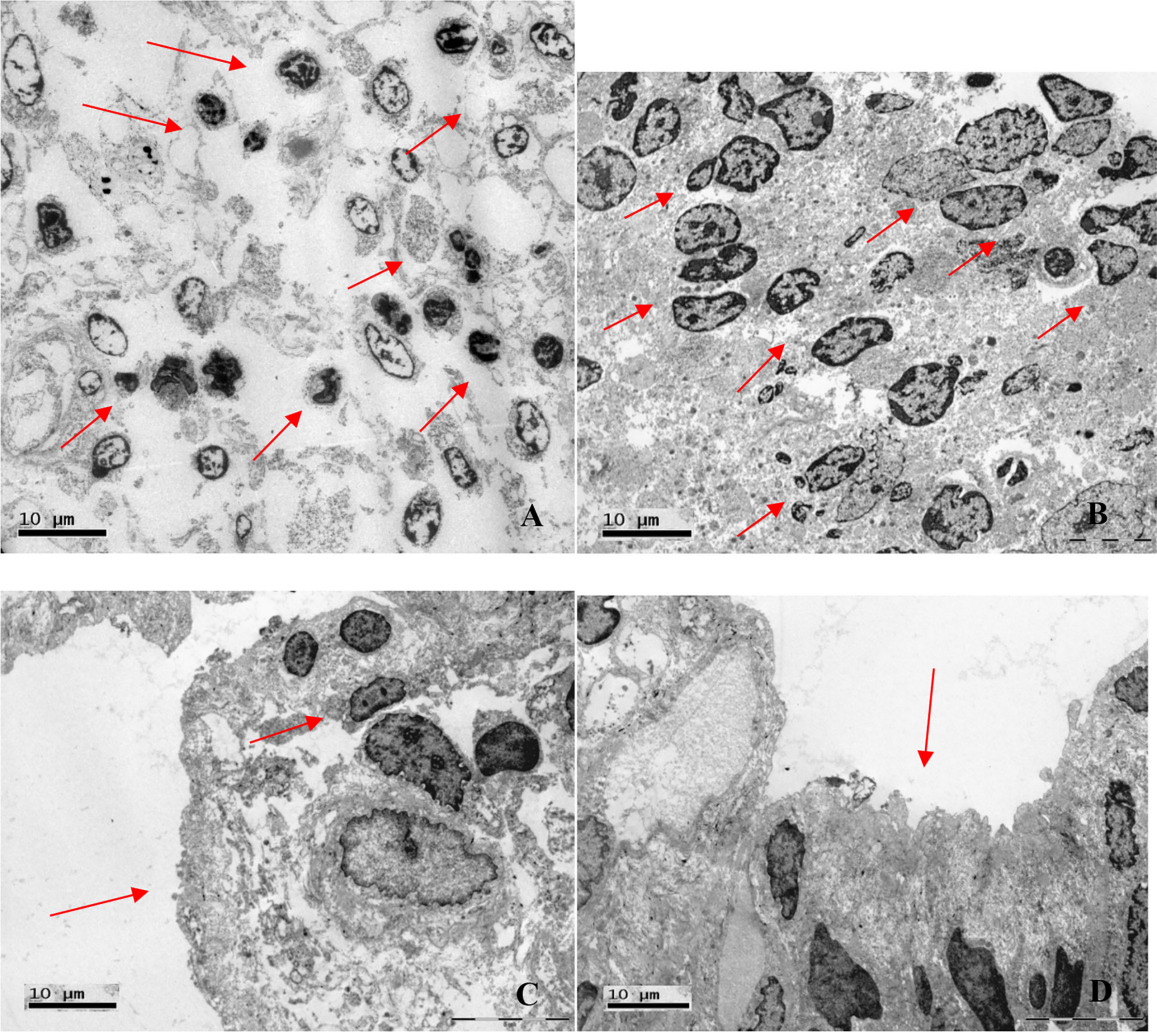
Electron photomicrographs of duodenal biopsies. **(A)** Lymphocytic inflammatory infiltrate (arrows; magnification, ×2,000). **(B)** Lymphocytic inflammatory infiltrate (arrows; magnification, ×2,550). **(C)** Mucosal atrophy and lymphocytic infiltration (arrows; magnification, ×3,700). **(D)** Microvillous atrophy (arrow; magnification, ×3,700).

Electron microscopy of the duodenum showed a mucosal lymphocytic inflammatory infiltrate in all four of the biopsy specimens analyzed and microvilli/mucosal atrophy in two (**Figure 6**). In two cases, neutrophils were also observed in the intestinal mucosa. The electron microscopy data are summarized in **Table 5**.

**Table 5:** Summary of the electron microscopy findings of liver and duodenal biopsies from CVID patients with liver disease/PH.

Patient	Liver findings	Duodenal findings
1	Relevant lymphocytic infiltration	Relevant lymphocytic infiltration
15	Mild lymphocytic infiltration	Relevant lymphocytic infiltration; vascular congestion
17	Relevant lymphocytic infiltration; hepatocyte apoptosis	Relevant lymphocytic infiltration; enterocyte apoptosis
37	Mild lymphocytic infiltration	Relevant lymphocytic infiltration; some neutrophils; microvillous atrophy

### β_2_-microglobulin in liver disease/PH

**Figure 7:**
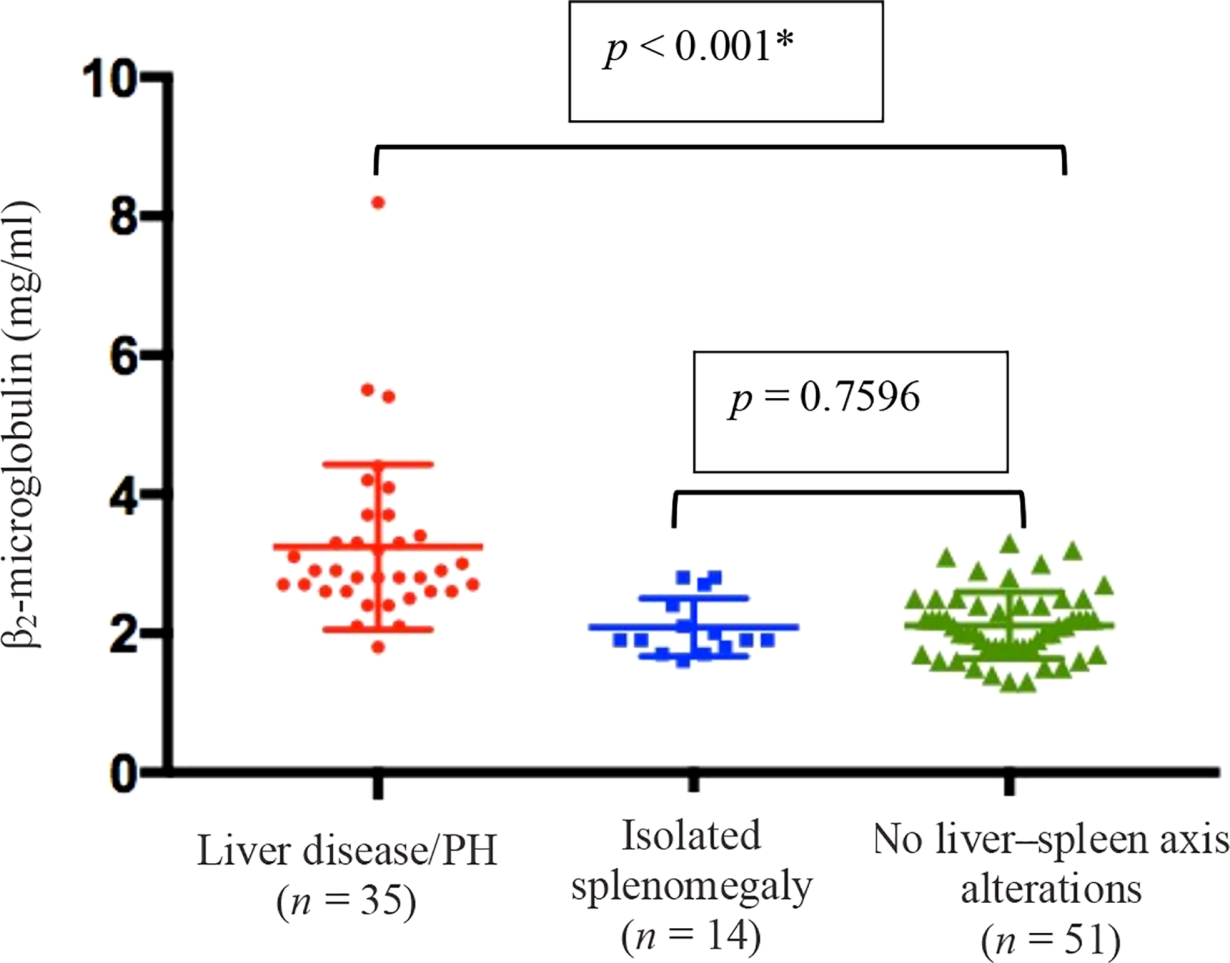
Means and standard deviations, together with individual patient values, for the level of β_2_-microglobulin in peripheral blood in the three groups of CVID patients evaluated (*n* = 100). ^†^ *Liver disease/PH vs. the other two groups (Kruskal–Wallis and Mann–Whitney *U* tests). ^†^Data unavailable for 41 patients.

**Figure 7** shows the serum levels of β_2_-microglobulin in all three groups. They were higher in the liver disease/PH group than in the other two groups (3.1 g/ml vs. 2.1 g/ml in the other groups; p < 0.001).

### B and NK lymphocytes in peripheral blood

As can be seen in **Table 6**, the mean quantity of B lymphocytes was lower in the liver disease/PH group than in the isolated splenomegaly group and the no liver–spleen axis abnormalities group (123.3 cells/mm^3^ vs. 134.5 cells/mm^3^ and 168.6 cells/mm^3^, respectively). The difference between the liver disease/PH group and the no liver–spleen axis abnormalities group was significant (p < 0.001). The mean NK-lymphocyte count was also lower in the liver disease/PH group than in the isolated splenomegaly group and the no liver–spleen axis abnormalities group (168.1 cells/mm^3^ vs. 163.4 cells/mm^3^ and 185.4 cells/mm^3^, respectively). Again, the difference between the liver disease/PH group and the no liver–spleen axis abnormalities group was statistically significant (p = 0.039). There were no statistically significant differences among the three groups in terms of the mean CD4^+^ and CD8^+^ T-lymphocyte counts (data not shown).

**Table 6:** Quantification of CD19^+^ and NK lymphocytes in the three groups of CVID patients evaluated (*n* = 137)*.

Group	CD19^+^	NK
	(cells/mm^3^)	(cells/mm^3^)
	Mean ± SD	Mean ± SD
Liver disease/PH	123.3 ± 194.3^†^	168.1 ± 194.1^‡^
Isolated splenomegaly	134.5 ± 118.2	163.0 ± 155.0
No liver–spleen axis abnormalities	168.6 ± 136.4	185.4 ± 139.6

SD, standard deviation.

*Data unavailable for four patients.

^†^ **p** < 0.001 vs. no liver–spleen axis abnormalities group.

^‡^ **p** = 0.039 vs. no liver–spleen axis abnormalities group.

### Liver disease/PH and lymphadenopathy

**Figure 8:**
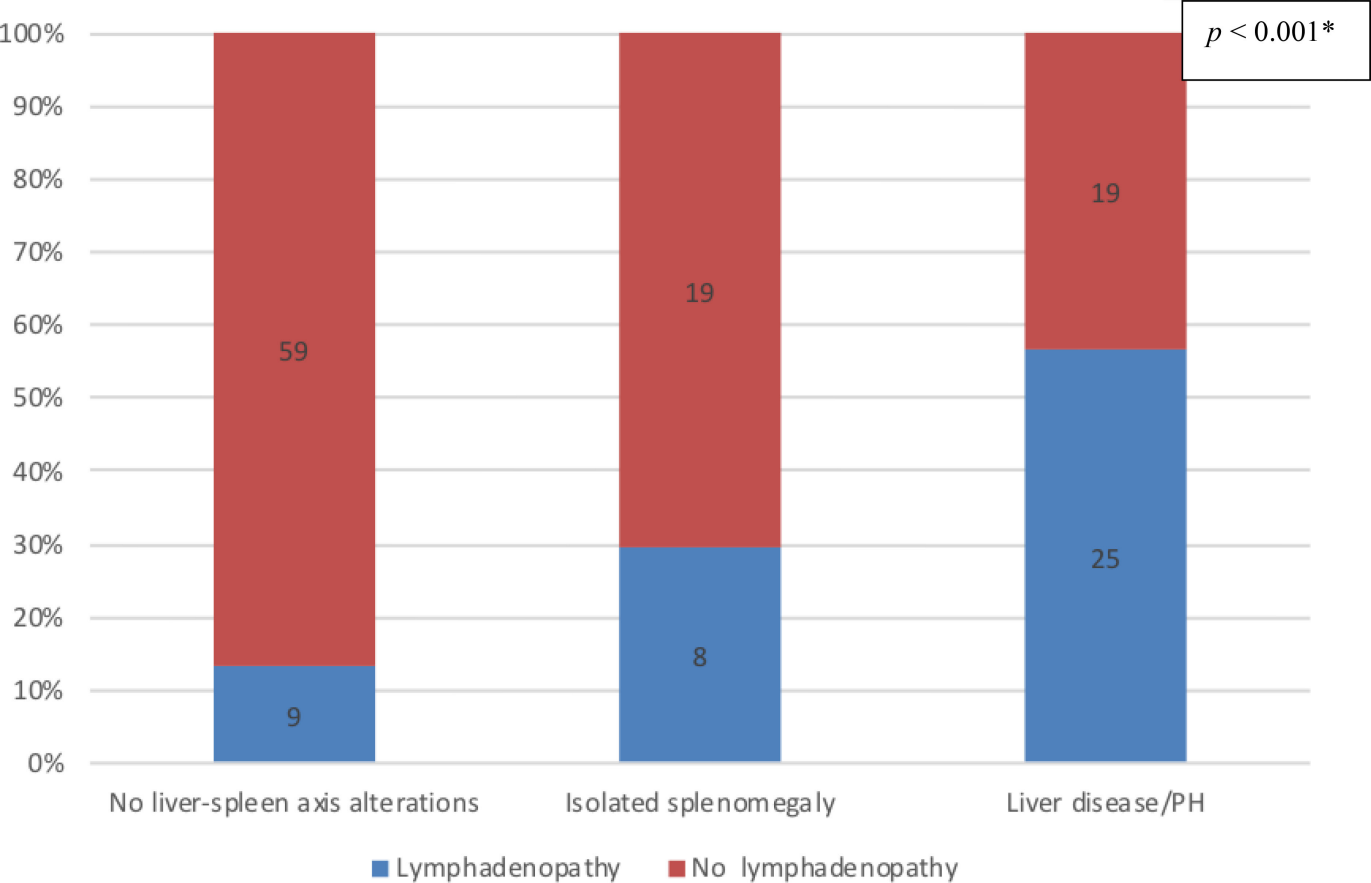
Prevalence of nonmalignant lymphadenopathy in the three groups of CVID patients evaluated (n = 139); no liver-spleen axis alterations n = 68; isolated splenomegaly n = 27; liver disease/PH n = 44. ^†^ *Versus the other two groups (Fisher’s exact test). ^†^Data unavailable for two patients.

Data regarding lymph node status were available for a total of 139 patients: 44 in the liver disease/PH group; 68 in the no liver–spleen axis abnormalities group; and 27 in the isolated splenomegaly group. Lymphadenopathy was present in 42 (30.2%) of the 139 patients analyzed: 25 (56.8%) of those in the liver disease/PH group; nine (13.2%) of those in the no liver–spleen axis abnormalities group; and eight (29.6%) of those in the isolated splenomegaly group. As illustrated in **Figure 8**, lymphadenopathy was significantly more prevalent in the liver disease/PH group than in the other two groups (p < 0.001 for both). There were no significant differences among the groups analyzed in terms of the presence of autoimmune disorders or bronchiectasis (data not shown).

### Liver disease/PH and mortality

During the study period, the mortality rate was highest in the liver disease/PH group, in which 12 (27.9%) of the 43 patients died, compared with three (12.5%) of the 24 patients in the isolated splenomegaly group and three (4.1%) of the 74 patients in the no liver–spleen axis abnormalities group (p < 0.01). However, none of the deaths were directly related to liver disease.

## Discussion

In patients with CVID, the occurrence of liver disease, which often manifests with signs of noncirrhotic PH, has been associated with increased morbidity and mortality (18). That is in keeping with our findings in the present study. Because there is as yet no specific treatment for liver disease, elucidation of the pathophysiology of liver injury, as well as that of other diseases associated with liver disease, could change the natural history of these complications in patients with CVID. Such knowledge might also be useful in the investigation of patients without CVID but with similar liver and intestinal lesions. Our detailed analysis of the characteristics of CVID patients who develop liver disease had the objective of supporting an active screening protocol for that complication. Our characterization of liver and duodenal lesions was aimed at looking for correlations between the two organs. To our knowledge, this is the first study to describe electron microscopy findings in liver biopsy specimens collected from CVID patients with liver disease and to suggest lymphocyte-mediated cytotoxicity against hepatocytes as the pathophysiological mechanism.

### Prevalence of liver disease/PH and splenomegaly in CVID

Pulvirenti et al. (16) studied a sample of 117 patients with primary hypogammaglobulinemia. The authors observed portal vein enlargement in 25.6% of the patients, although the frequency of isolated splenomegaly they reported (35.0%) was higher than the 19.1% observed in our study. That could be attributed to a difference between the two studies in terms of the manifestations of CVID in the respective study populations. In a study of 77 CVID patients evaluated with liver elastography, Crescenzi et al. (30) found that alterations indicative of fibrosis, which can be accompanied by PH, were present in 33.8%, a proportion similar to that observed in our liver disease/PH group. In two other studies of patients with CVID, the reported prevalence of splenomegaly was 30% (7) and 26% (13). However, neither of those studies included data regarding the size of the portal vein or the presence of associated liver disease. In comparison with our data, Azzu et al. (18), Malamut et al. (41), and Ward et al. (17) found a much higher prevalence of liver disease in CVID patients—79%, 54%, and 44%, respectively—although those studies applied broader criteria to define liver disease, such as the alteration of at least one hepatic or canalicular enzyme. Malamut et al. (41) identified PH in 50% of the patients with primary hypogammaglobulinemia (42.5% of those with CVID), a prevalence higher than that seen in our cohort. Azzu et al. (18) found that 54% of their CVID patients had liver disease together with splenomegaly and that 22% had splenomegaly alone.

### Clinical and biochemical characteristics of CVID patients with liver disease/PH

In the present study, the mean age of the patients was significantly higher in the liver disease/PH group than in the other groups. In contrast, Azzu et al. (18) observed no age difference between CVID patients with and without liver disease, although those authors applied different selection criteria. Ward et al. (17) found no significant associations among the different groups of patients regarding age at symptom onset, age at CVID diagnosis, or duration of disease, findings that corroborate those of the present study. These data suggest that there is a subgroup of patients in which the evolution to liver disease is part of the natural history of CVID, possibly associated with a specific immunological phenotype. An early age at CVID symptom onset has been associated with a higher frequency of airway infections (3). Conversely, the late onset of CVID has been associated with autoimmune disorders, neoplasia, and splenomegaly (3). None of those variables have been found to be associated with liver disease, either in our study or in others (16, 17, 20).

The fact that most of the patients with liver disease/PH in our study also had splenomegaly and presented with only mild or even no biochemical abnormalities illustrates the need for an evaluation protocol that includes regular imaging examinations to uncover other evidence of liver disease and PH in patients with CVID. Most of our liver disease/PH group patients had low platelet counts, which were not observed among the patients in the other groups. Thrombocytopenia, caused by splenic sequestration, is a common complication in patients with PH (16). However, in CVID, thrombocytopenia can also be an autoimmune disorder. Azzu et al. (18) observed thrombocytopenia in only 32% of their patients with liver disease, compared with 63% of those in the present study. In a study of patients with primary hypogammaglobulinemia and liver abnormalities, Malamut et al. (20) found elevated hepatic transaminases in 49% of cases and cholestasis in 65%, compared with approximately 35% and 22%, respectively, in the present study. Again, the discrepancies between our findings and those of previous studies are likely attributable to differences in the criteria applied for the diagnosis of liver disease.

Complications such as esophageal and gastric varices were observed in approximately half of the patients evaluated in the present study and can occur in the absence of relevant changes in transaminases, canalicular enzymes, bilirubin, platelet counts, or liver function. Often, in the early stages of liver disease, liver function remains normal even in the presence of esophageal varices and splenomegaly (49). Pulvirenti et al. (16) found esophageal varices in 3.4% of patients with primary hypogammaglobulinemia, much lower than the 39.1% observed in our sample of CVID patients. However, Malamut et al. (41) reported the prevalence of esophageal varices to be approximately 20% in patients with liver disease. Those authors documented liver failure (defined as a 50% increase in prothrombin time) in three of the 51 patients evaluated, compared with two of the 46 patients evaluated in the present study. The high variability across studies might be due to factors inherent to the patient samples, such as CVID features, degree of immune dysregulation, or even genetic background.

### Clinical and immunological features of liver disease and PH in CVID

Among the patients evaluated in the present study, nonmalignant lymphadenopathy was observed in 30.2%, most of whom were in the liver disease/PH group. Lymphadenopathy might be involved in the pathophysiology of PH and liver disease in CVID. It has been reported that NRH is a cause of PH in hematological diseases (50), which is supported by the fact that indolent proliferation of cytotoxic T cells is associated with NRH (51). In two other studies (17, 30), a significant relationship was detected between liver disease and lymphadenopathy. As has previously been demonstrated (52), β_2_-microglobulin is a low-molecular-weight protein released by activated lymphocytes, tumor cells, and other cells. It is a marker of activity in inflammatory diseases (53) and can be elevated in liver disease (54). North et al. (55) found that high levels of β_2_-microglobulin correlated with the severity of the B-lymphocyte defect as well as with the severity of CVID. In the present study, we found that serum levels of β_2_-microglobulin were highest in the liver disease/PH group.

In our study, B- and NK-lymphocyte counts were lowest in the liver disease/PH group, which suggests a distinct immunological phenotype in such patients. Changes in specific lymphocyte subpopulations have been observed in CVID patients with the non-infectious phenotype (56). Malamut et al. also reported low B-lymphocyte counts (<70 cells/mm^3^) in CVID patients. However, it has also been reported that CD4^+^ T-cell counts are low (<400 cells/mm^3^) in CVID patients with liver disease (41). In the present study, we found that autoimmune disorders showed no relationship with liver disease, PH, or splenomegaly, a finding corroborated by Ward et al. (17). In addition, as previously demonstrated by Azzu et al. (18), we detected no correlation with bronchiectasis.

### Mechanism of liver injury and its relationship with PH in CVID

We identified NRH in the liver biopsies of all 11 patients with CVID and PH who underwent the procedure. As described in the literature (17, 19–21), NRH is the main liver disorder observed in CVID patients, as well as having been associated with connective tissue diseases, Crohn’s disease, chronic viral infections, exposure to medications, and prothrombotic conditions. However, the cause of NRH remains unclear, and it seems to be a secondary complication (44). Although the diagnosis of autoimmune hepatitis in CVID patients is challenging because of the impaired antibody production, the characteristic changes, such as marked interface hepatitis and apoptotic hepatocytes, were not observed in the liver biopsies evaluated in the present study. Therefore, an autoimmune hepatitis-like mechanism is unlikely.

As observed in our patient sample, NRH is known to be associated with PH (17–20). In another study of CVID patients, Crotty et al. (57) diagnosed PH in 75% of those who underwent liver biopsy. In that study, there was also mild-to-moderate portal inflammation in 75% of the patients and lobular inflammation in approximately 70%. The authors identified NRH in approximately 92% of the biopsies and sinusoidal mononuclear cells (mimicking the lymphocytic infiltration of Epstein–Barr virus infection) in 54.2%. As in our study, the liver function test results in that study were slightly abnormal, especially ALP and AST. The classic features of primary biliary cholangitis, which was not observed in our sample, were observed in two of the biopsies evaluated by those authors (57). Other studies have shown that, in patients with NRH but without CVID, the changes in transaminases and canalicular enzymes are modest, highlighting ALP, which can present a progressive increase even when liver function is preserved (25, 58). Another study identified NRH in the biopsies of 12% of the CVID patients evaluated (17), although the authors suggested that the prevalence might have been underestimated, given that NRH can occur in the absence of relevant biochemical abnormalities, which corroborates our data.

In a study of patients with hypogammaglobulinemia and liver disease (20), NRH was diagnosed in 87% of the patients who underwent biopsy and was accompanied by PH in 74%. The authors observed no significant portal fibrosis/cirrhosis or hepatocyte necrosis (except in a patient with hepatitis C). A moderate or significant sinusoidal inflammatory infiltrate, composed mainly of CD3^+^CD8^+^ lymphocytes, was observed in 87% of the patients evaluated in that study. In the same study, non-necrotizing, non-fibrosing granulomas were detected in approximately 44% of the patients who underwent biopsy, and portal endotheliitis was detected in 35%. In the present study, the hepatic pathology findings were similar to those of that study, except for those related to the granulomas: in our sample, we observed nodular formations together with a mild-to-moderate periportal inflammatory infiltrate, composed mainly of mononuclear cells.

In a study of nine patients with CVID, Fuss et al. (19) observed increased pressure in the portal vein, mild changes in liver enzymes (2–3× the reference value), thrombocytopenia, and NRH. As in our study, their pathology analysis identified nodular regeneration, together with perisinusoidal fibrosis and irregular lobular lymphocytic inflammatory foci, albeit in only three of the patients evaluated. In the remaining patients, the authors observed mild-to-moderate focal portal inflammatory infiltrates, without necrosis, except in one patient, who presented with extensive bridging portal fibrosis. In all nine patients, few B lymphocytes were identified (most cells in the infiltrate were CD8^+^ T cells), being present in the sinusoids in approximately one third of the patients; the B lymphocytes were clonal and granzyme B+, which is suggestive of specific liver cytotoxicity. Also, as in our study, cells indicative of activated macrophages (Kupffer cells) were found. The authors suggested that a specific group of five patients, who also had a pathological diagnosis of NRH, had an autoimmune hepatitis-like liver disease. The features of NRH were accompanied by portal inflammatory infiltration and bridging necrosis, as well as (in one patient) prominent bridging periportal and perisinusoidal fibrosis, characteristics that were not found in our study sample. However, the authors argued that those alterations might constitute a form of NRH that is more severe than is typical autoimmune hepatitis. In addition, those patients presented with a clinical picture distinct from that observed in our patients and that of classical NRH, including severe hepatitis, together with impaired hepatic excretion and synthesis. Two of those five patients were treated with immunosuppressants. However, those two patients subsequently died of progressive liver disease and associated systemic infection. In one patient, the disease was successfully controlled with steroids and an anti-metabolite (6-mercaptopurine).

Despite the difficulty of diagnosing autoimmune hepatitis in CVID, liver biopsy findings can support the diagnosis, even without serum liver autoantibodies, in some cases. In such cases, the diagnosis is made on the basis of the presence of interface hepatitis, plasma cells, and hepatocyte rosettes (59), none of which were observed in our sample. In two case reports of patients with CVID and autoimmune hepatitis (60, 61), the changes in aminotransferases, bilirubin, and liver function were much more severe than those seen in NRH. In the case described in the first report (60), AST and ALT levels were 47× and 31× higher than the reference values, respectively, whereas ALP and total bilirubin were 5× and 8× higher, respectively, with an international normalized ratio of 3.1 and a serum albumin level of 2.5 g/L. In the case described in the second report (61), AST, ALT, ALP, and GGT levels were 44×, 9×, 2×, and 6× higher than the reference values, respectively. In both cases, abdominal imaging revealed a cirrhotic-appearing liver, which does not occur in NRH, and the liver biopsy findings were consistent with autoimmune hepatitis in both cases. In the first report (60), the patient had prominent lymphocytic portal and lobular inflammation, numerous mature lymphocytes and plasma cells, typical interface hepatitis, and stage 3 (septal) fibrosis. In the second report (61), the patient had severe hepatocellular injury with confluent necrosis, parenchymal necrosis, lobular inflammation suggestive of acute hepatitis, lymphocytic infiltration with interface activity, and plasma cells. In both cases, there was a good clinical response to immunosuppressive therapy. Drawing a parallel with seronegative autoimmune hepatitis (62), one can also establish the diagnosis on the basis of the characteristic pathology findings, together with a good response to immunosuppressive therapy, which plays an important role in such cases, in which the pathophysiology appears to be distinct from that of NRH.

Our electron microscopy findings support the hypothesis that the mechanism of injury to hepatocytes is, in fact, lymphocytic infiltration. The images suggest lymphocyte-mediated cytotoxicity against hepatocytes, resulting in liver damage. The reason for this phenomenon remains unknown. In CVID, lymphocytic infiltration can be seen in various organs, probably as a sign of immune dysregulation (7, 13). As previously discussed, NRH is associated with secondary immunodeficiency (caused by neoplasms or the use of immunosuppressants), primary immunodeficiency (CVID), and viral infections. It is likely that the activation of cytotoxic lymphocytes is secondary to an infection. Another possibility is chronic immune activation in the liver and intestinal circulation as a consequence of recurrent infection (63). It is well known that chronic immune activation is common in CVID, as described in a previous study conducted by our group (64).

### Liver disease/PH and duodenal celiac pattern in CVID

In the present study, a duodenal celiac pattern was found in 14.4% of the biopsy specimens, mostly from patients in the liver disease/PH group. Chapel et al. (7) and Cunningham-Rundles et al. (36) identified a duodenal celiac pattern in 8% and 2% of CVID patients, respectively. The difference between their findings and ours might be due to environmental factors in the different study populations, such as the presence of intestinal pathogens, which can also cause intraepithelial lymphocytosis.

Among the 11 patients with PH and NRH in our sample, duodenal mucosal atrophy was identified in three cases and an inflammatory infiltrate (mainly mononuclear and of varying intensity) was identified in all but one case. In fact, the duodenal celiac pattern is common in CVID patients with enteropathy. In patients with CVID and gastrointestinal symptoms, the reported prevalence of duodenal intraepithelial lymphocytosis ranges from 17.1% to 75.6% and that of villous blunting ranges from 24.4% to 53.0% (39, 41, 65). Malamut et al. (41) observed that intraepithelial lymphocytes in CVID patients were predominantly CD3^+^ and CD8^+^. Differences in the genetic and environmental characteristics of the patient samples could account for the discrepancies between our findings and those in the literature.

Two other studies have also reported a statistically significant relationship between liver disease and celiac-like enteropathy in CVID, suggesting that there is common pathophysiology (17, 30). Noncirrhotic PH can be secondary to mechanisms of immune stimulation by pathogens in the portal vein (63, 66) and consequent T-lymphocyte proliferation (67). There have been case reports describing NRH in combination with celiac disease (68). The relationship that duodenal celiac pattern has with noncirrhotic PH and liver disease appears to be similar to that observed in patients with CVID. These results are in keeping with those of Daniels et al. (69), who reported an intestinal inflammatory infiltrate indicative of duodenal celiac pattern in five of seven patients with CVID who had elevated liver enzymes and hepatomegaly, with or without splenomegaly, and underwent liver biopsy. In CVID, a duodenal celiac pattern and the activation of cytotoxic lymphocytes can be secondary to infection.

Enteropathy with a duodenal celiac pattern in CVID differs from that occurring in celiac disease in that it rarely improves with gluten exclusion (36, 70, 71). Infiltration by intraepithelial T lymphocytes might represent a compensatory but ineffective defense mechanism. In patients with hypogammaglobulinemia, villous atrophy has been associated with an increase in T-cell receptor γδ+ intraepithelial T lymphocytes, whereas T-cell receptor αβ+ intraepithelial T lymphocytes are increased in celiac disease (41). Infectious agents have been reported in association with enteropathy in CVID. In a CVID patient with severe acute duodenitis, Malamut et al. (41) identified *G. lamblia* and evidence of previous chronic infection with *C. jejuni, Cryptosporidium* sp., and *Clostridioides difficile*. Analyzing 44 CVID patients with gastrointestinal symptoms, van Schewick et al. (65) identified *C. jejuni* together with norovirus infection in four patients, *C. difficile* in three, *G. lamblia* in two, and cytomegalovirus in one, although they did not screen for infectious agents in all of the patients. In CVID, norovirus infection can also cause duodenal atrophy, lymphocytic infiltration, and duodenal celiac pattern (72). In immunocompromised individuals, norovirus infection leads to severe, prolonged disease, together with chronic diarrhea (73–75). Proper identification of the causative agent of enteropathy is essential to understanding pathological intestinal alterations, guiding patient treatment, and providing insights for the clarification of gut–liver axis pathologies.

In the present study, we found that mortality rates were higher among the CVID patients with liver disease or PH than among those with neither. Resnick et al. (3) reported that the risk of death was 2.48 times higher in CVID patients with liver disease than in those without (3), which is comparable to what we observed in our sample. In another study (18), the crude mortality rates for CVID patients with and without liver disease were 28% and 6%, respectively. This worse prognosis might be related to the pathophysiology that results in liver damage, together with complications such as lymphadenopathy and duodenal celiac pattern. Therefore, it is of fundamental importance to monitor CVID patients, through the use of regular liver function laboratory tests in conjunction with imaging examinations, for signs of PH or parenchymal liver disease, given that liver disease/PH is probably underdiagnosed in such patients.

In the present study, we have provided a detailed analysis of the clinical, biochemical, and histopathological characteristics of CVID patients and liver disease in a large patient sample in Brazil. Electron microscopy of the liver, performed for the first time in CVID patients, corroborated the histopathological findings and suggested that the mechanism of hepatocyte injury is in fact associated with lymphocytic infiltration, especially of hepatocytes. Although there is as yet no specific treatment for liver disease in CVID patients, elucidation of the mechanism of liver injury, associated pathologies, and probable immune dysregulation is crucial to understanding the cause of this complication and enabling the development of therapeutic options for the affected patients.

## Conclusions

We found the prevalence of liver disease, leading to PH, to be high in a subgroup of CVID patients, in whom it had a direct impact on mortality. That underscores the importance of adequate screening for that complication. Liver damage and PH can occur in CVID, even in patients with mild or no biochemical abnormalities. It is therefore important to perform imaging examinations to look for signs such as portal vein enlargement, splenomegaly, esophageal varices, and collateral circulation.

Patients with CVID and liver disease/PH have lower B- and NK-lymphocyte counts, which suggests that there is a distinct immunological phenotype that is more prone to developing that complication. One marker of that clinical phenotype is β_2_-microglobulin, the elevation of which is also a predictor of morbidity and mortality in CVID.

The main mechanism of liver damage in CVID is NRH. In patients with liver disease/PH, a pathology study is essential for the diagnosis and early treatment of associated conditions such as esophageal varices.

Lymphadenopathy and duodenal celiac pattern are strongly associated with PH, which supports the concept of a specific CVID phenotype and suggests that the pathophysiology of these conditions have a common mechanism. The pathological and electron microscopy findings of NRH-related lymphocytic infiltration of the duodenum indicates that the pathophysiologies of these two conditions are similar. Electron microscopy findings suggest that the mechanism of liver injury is lymphocyte-mediated cytotoxicity against hepatocytes.

Patients with CVID and liver disease/PH have a distinct immunological phenotype that may predispose to liver and duodenal lymphocyte-mediated cytotoxicity. Further studies are required to better know the cause of this immune mediated mechanism and its treatment options.

## Data availability statement

The original contributions presented in the study are included in the article/**Supplementary Material**. Further inquiries can be directed to the corresponding author.

## Ethics statement

The studies involving human participants were reviewed and approved by Research Ethics Committee of the Hospital das Clínicas, Faculdade de Medicina, Universidade of São Paulo (HC-FMUSP). Written informed consent for participation was not required for this study in accordance with the national legislation and the institutional requirements.

## Author contributions

FL, CK, MT-B, and JK conceived of the study design. FL performed the data collection. VA and MD led the anatomopathological analysis. MD led the electron microscopy analysis. CT contributed to the electron microscopy. FL, CK, and MT-B performed the data analysis. JK, CS, and AM contributed to the data analysis. FL, CK, MT-B, VA, and OG drafted the manuscript. All authors verified the analytical methods, discussed the results, read and approved the final manuscript.

## Funding

We are grateful for the support received from the Brazilian Conselho Nacional de Desenvolvimento Científico e Tecnológico (CNPq, National Council for Scientific and Technological Development); Instituto Nacional de Ciência e Tecnologia de Investigação em Imunologia (National Institute of Science and Technology for Investigation in Immunology-III/INCT), Sao Paulo, SP, Brazil; Conselho Nacional de Desenvolvimento Científico e Tecnológico (National Council for Scientific and Technological Development) - CNPq – Brazil; Project INCT/CNPq-iii; Process number 465.434/2014-2.

## Conflict of interest

The authors declare that the research was conducted in the absence of any commercial or financial relationships that could be construed as a potential conflict of interest.

## Publisher’s note

All claims expressed in this article are solely those of the authors and do not necessarily represent those of their affiliated organizations, or those of the publisher, the editors and the reviewers. Any product that may be evaluated in this article, or claim that may be made by its manufacturer, is not guaranteed or endorsed by the publisher.
