# Supplementary material for: Liver disease accompanied by enteropathy in common variable immunodeficiency: Common pathophysiological mechanisms
Source: Front Immunol. 2022 Oct 20;13:933463. doi: 10.3389/fimmu.2022.933463 (PMC9632424; doi:10.3389/fimmu.2022.933463)
Supplement: Supplementary file 1 [file Table_1.docx]

Table S1. Clinical and laboratory characteristics of 46 patients diagnosed with liver disease/portal hypertension (PH).

|  | Complications of liver disease | AST  (x RV) | ALT  (x RV) | ALP  (x RV) | GGT  (x RV) | TB  (x RV) | DB  (x RV) | INR  (x RV) | platelets (10^3^/mm³) |
| --- | --- | --- | --- | --- | --- | --- | --- | --- | --- |
| Patient 1 | ascites  hepatomegaly  splenomegaly | 1.5x | 3.1x | 3x | 3.4x | NL | NL | 1 | 105 |
| Patient 2 | hepatomegaly  splenomegaly | NL | NL | NL | 1.5x | NL | NL | 0.96 | 77 |
| Patient 3 | none | NL | 1.2x | NL | 1,2x | NL | NL | 1.1 | 153 |
| Patient 4 | collateral circulation | NL | NL | 1.3x | NL | 1.1x | 1.1x | 0.95 | 94 |
| Patient 5 | esophageal varices  gastric varices  collateral circulation  ascites  splenomegaly | 2.4x | 2.2x | 2.8x | NL | NL | 1.9x | 1.29 | 75 |
| Patient 6 | hepatomegaly  splenomegaly | NL | NL | NL | NL | NL | NL | 1.09 | 123 |
| Patient 7 | esophageal varices  ascites  splenomegaly | 1.3x | NL | 2.3x | 1.8x | NL | NL | 1.02 | 59 |
| Patient 8 | splenomegaly | NL | NL | 2.9x | 1.6x | NL | NL | NA | 48 |
| Patient 9 | esophageal varices  gastric varices  splenomegaly | NL | NL | NL | NL | NL | NL | 0,95 | 66 |
| Patient 10 | splenomegaly | NL | NL | 1.5x | 1.5x | NL | NL | 1 | 215 |
| Patient 11 | esophageal varices  gastric varices  hypertensive colopathy  splenomegaly | NL | NL | 2.7x | 3.4x | NL | NL | 1.09 | 225 |
| Patient 12 | hepatomegaly  splenomegaly | NL | NL | 1.1x | 1.1x | NL | NL | 1.04 | 122 |
| Patient 13 | esophageal varices  splenomegaly | NL | NL | 1.3x | 4x | NL | NL | 2.73 | 33 |
| Patient 14 | liver-spleen shunt  splenomegaly | NL | NL | NL | NL | NL | NL | 1 | 209 |
| Patient 15 | esophageal varices  gastric varices  perisplenic varices  splenomegaly | NL | NL | 1.4x | NL | NL | NL | 1.16 | 126 |
| Patient 16 | splenomegaly | NL | NL | NL | 1.3x | NL | NL | 1.04 | 98 |
| Patient 17 | esophageal varices  splenomegaly | 2.8x | NL | 5.1x | 6.8x | NL | 1.7x | 1.1 | 269 |
| Patient 18 | collateral circulation splenomegaly | NL | NL | NL | NL | NL | NL | 1.02 | 202 |
| Patient 19 | splenomegaly | 1.3x | 1.2x | 1.6x | 2.5x | NL | NL | 1.05 | 134 |
| Patient 20 | splenomegaly | NL | NL | NL | NL | NL | NL | 1.06 | 100 |
| Patient 21 | splenomegaly | 1.5x | 1.4x | 1.3x | 6.6x | NL | 1.6x | 1.18 | 122 |
| Patient 22 | esophageal varices  collateral circulation | NL | NL | NL | 5x | NL | NL | 1.02 | 339 |
| Patient 23 | esophageal varices  gastric varices  splenomegaly | NL | 1,6x | 2x | NL | NL | 1,8x | 1,65 | 228 |
| Patient 24** | gastric varices  splenomegaly | 2.6x | 1.3x | 1.4x | 4.7x | 5x | 16.3x | 1.2 | 296 |
| Patient 25 | esophageal varices  gastric varices  splenomegaly | NL | NL | 1.6x | 2.3x | NL | NL | 0.95 | 107 |
| Patient 26 | splenomegaly | NL | NL | NL | NL | NL | NL | 1 | 110 |
| Patient 27 | hepatomegaly  splenomegaly | 2.6x | 2.6x | 3x | 2x | NL | NL | 0,99 | 57 |
| Patient 28 | enlargement of the splenic vein | NL | NL | NL | NL | NL | NL | 1.04 | 126 |
| Patient 29 | splenomegaly | NL | NL | NL | NL | NL | NL | 0.95 | 190 |
| Patient 30 | splenomegaly | NL | NL | NL | NL | NL | NL | 0.95 | 168 |
| Patient 31 | splenomegaly | 2.1x | NL | 1.1x | NL | NL | NL | 1.33 | 110 |
| Patient 32 | splenomegaly | 1.4x | 1.4x | NL | NL | NL | NL | 1.31 | 189 |
| Patient 33 | esophageal varices  ascites | 2.1x | 1.9x | 2.2x | 1.8x | NL | NL | 1.29 | 123 |
| Patient 34 | esophageal varices  splenomegaly | NL | NL | 1.2x | NL | NL | NL | 0.94 | 91 |
| Patient 35 | esophageal varices  hepatomegaly | 1.7x | 1.9x | 3.2x | 4.1x | NL | NL | 1.15 | 94 |
| Patient 36 | esophageal varices  gastric varices | NL | NL | 4x | 5.4x | NL | NL | 0.94 | 241 |
| Patient 37** | gastric varices  splenomegaly | 1.7x | 1.1x | 3.9x | 5.6x | 1.2x | 1.9x | 0.95 | 72 |
| Patient 38 | esophageal varices  splenomegaly | 2.1x | 1.6x | 4x | 2.8x | 1.1x | 1.7x | 0.5 | 69 |
| Patient 39 | splenomegaly | NL | NL | NL | 2.9x | 2.9x | 2.8x | 1.07 | 96 |
| Patient 40 | esophageal varices  collateral circulation  splenomegaly | NL | NL | 1.1x | 2.4x | NL | 1.8x | 1.16 | 125 |
| Patient 41 | esophageal varices  splenomegaly | 1.3x | 1.2x | 3.4x | 4.5x | NL | NL | 1.19 | 67 |
| Patient 42 | splenomegaly | NL | NL | NL | NL | NL | NL | ND | 276 |
| Patient  43 | splenomegaly | NL | NL | NL | NL | NL | NL | 1.2 | 185 |
| Patient 44 | ascites  splenomegaly | NL | NL | 1.2x | NL | NL | NL | 1 | 287 |
| Patient 45 | esophageal varices  splenomegaly | 1.2x | 1.2x | 2.3x | 1.8x | NL | NL | 1.1 | 51 |
| Patient 46** | none | NL | NL | NL | NL | NL | NL | 0.95 | 575 |

# Reference Values (RV): Liver enzymes: alanine aminotransferase (ALT) <41 U/L for men and <31 for women, aspartate aminotransferase (AST) <37 U/L for men and <31 for women. Alkaline Phosphatase (ALP) - 40 to 129 U/L for men and 35-104 U/L for women, gamma glutamyl transferase (GGT) - 8 to 61 U/L for men and 5-36 U/L for women. Total bilirubin (BT) - 0.20 to 1.00 mg/dL, direct bilirubin (BD) - 0.30 mg/dL, indirect bilirubin (BI) - 0.10 to 0.60 mg/dL. INR: 0.95 to 1.20. Platelets: 140 - 450 103/mm³. Values shown according to the increase above the reference values. **Patients 24 and 37 were diagnosed with non-Hodgkin's lymphoma. All had evidence of PH before the diagnosis of neoplasia. Patient 46 underwent splenectomy due to external cause.
